# Hispanic-White Differences in Double Bonuses for Quality of Care in Medicare Advantage

**DOI:** 10.1001/jamahealthforum.2021.5281

**Published:** 2022-03-04

**Authors:** Adam A. Markovitz, Marie C. Montás, Anupama Warrier, John Z. Ayanian, Andrew M. Ryan

**Affiliations:** 1 Department of Internal Medicine, University of Michigan Medical School, Ann Arbor; 2 Department of Health Management and Policy, School of Public Health, University of Michigan, Ann Arbor; 3 University of Michigan Center for Evaluating Health Reform, Ann Arbor; 4 University of Michigan Institute for Healthcare Policy and Innovation, Ann Arbor

## Abstract

This study uses Medicare and Medicare Advantage data to describe differences in quality care between Hispanic and non-Hispanic White patients and the association with double bonuses under Medicare Advantage.

## Introduction

The growth of Medicare Advantage (MA), in which beneficiaries enroll in private health plans, has been remarkably rapid for Hispanic enrollees, increasing from 33% to 48% from 2009 through 2018.^1^ However, evidence suggests that Hispanic enrollees may receive lower quality of care than their non-Hispanic White counterparts.^2^ The structural drivers of these disparities are myriad, including immigration policy, health literacy, and the COVID-19 pandemic.^3^ One underexplored driver may be disparities in health care financing. Since 2012, high-quality MA plans have received financial bonuses under the Quality Bonus Program. In “double-bonus” counties with high MA enrollment and low fee-for-service spending, high-quality plans receive bonuses twice as large as those in non–double-bonus counties. Double bonuses totaled $10.2 billion from 2012 through 2018, yet prior research has shown that they do not improve quality of care and are offered less frequently to Black enrollees than to White enrollees.^4^ We examined whether Hispanic enrollees were less likely than White enrollees to reside in counties eligible for double bonuses and, if so, whether this reduced payments to plans caring for Hispanic enrollees.

## Methods

First, using 2012-2019 Medicare Beneficiary Summary Files and MA ratebook data, we estimated the likelihood of residing in a double-bonus county among Hispanic and White enrollees via a serial cross-sectional logistic regression model estimated at the enrollee-year level, controlling for RTI race and ethnicity code (91% sensitive and 99% specific for Hispanic ethnicity^5^), age, sex, reason for Medicare entitlement, Medicaid dual eligibility, and year fixed effects (eMethods and eFigure in the Supplement). Given the paucity of data on Hispanic payment disparities in Medicare, we focused on Hispanic and White MA enrollees but used analogous methods from prior work.^4^ Second, we estimated mean payments to plans in double-bonus and non–double-bonus counties via a linear regression model estimated at the plan-year level, weighted by enrollment, and adjusted for plan star rating, county-specific benchmark payments, and year fixed effects. Finally, we calculated Hispanic-White payment disparities by multiplying the Hispanic-White difference in residing in a double-bonus county (step 1) by the difference in payments to plans in double-bonus vs non–double-bonus counties (step 2). The University of Michigan institutional review board exempted our study from review because it used deidentified administrative data. This cross-sectional study followed the Strengthening the Reporting of Observational Studies in Epidemiology (STROBE) reporting guideline. Our analyses were performed in June 2021 and used Stata, version 16 (StataCorp LLC).

## Results

MA enrollees contributed 113 876 310 enrollee-years, comprising 12.8% Hispanic and 87.2% White enrollees (Table 1). Hispanic enrollees were younger and more likely to be dual eligible for Medicaid and entitled to Medicare owing to disability or end-stage kidney disease.

**Table 1.** Characteristics of Hispanic and Non-Hispanic White MA Enrollees, 2012 to 2019

Characteristic	MA enrollees, mean (SD)^a^^,^^b^		Standardized difference
	Hispanic	White	
Sample, No.			
Enrollee-years	14 589 670	99 286 640	NA
Enrollees	3 137 324	20 839 780	NA
Age, y	71.7 (10.7)	73.5 (9.9)	−0.17
Sex, %			
Female	55.6 (49.7)	56.3 (49.6)	−0.01
Male	44.4 (49.7)	43.7 (49.6)	0.01
Original Medicare entitlement due to disability or end-stage kidney disease, %	26.7 (44.2)	19.8 (39.9)	0.16
Medicaid dual eligibility, mo	4.7 (5.7)	1.3 (3.6)	0.72

Abbreviations: MA, Medicare Advantage; NA, not applicable.

^a^ Enrollee race and ethnicity was based on the RTI race code, which supplements self-reported data with lists of Hispanic surnames from US Census data and residence in Puerto Rico.^6^

^b^ Defined using January enrollment status.

Hispanic enrollees were 11.8 percentage points less likely than White enrollees to reside in a double-bonus county (17.4% vs 29.2%, respectively; difference, 95% CI, −19.5% to −4.0% percentage points) (Table 2). Mean payments were $320 higher for plans in double-bonus vs non–double bonus counties (eAppendix in the Supplement). Taken together and holding quality performance constant across plans, the double-bonus policy increased mean plan payments by $56 for Hispanic enrollees and by $93 for White enrollees, a disparity of $38 per enrollee per year (Table 2). In aggregate, White MA populations gained $551 million more than Hispanic populations from 2012 through 2019 through double bonuses.

**Table 2.** Association of Double-Bonus Policy With Payments for MA Plans to Care for Hispanic and Non-Hispanic White Enrollees^a^

Statistic	MA enrollees		Hispanic-White disparity
	Hispanic	White	
Likelihood of an individual MA enrollee residing in a double-bonus county	17.4%	29.2%	Percentage point, −11.8 (95% CI, −19.5 to −4.0)
Incremental difference in mean payment to MA plans associated with the double-bonus policy	$56 Per enrollee-year (0.5% mean total payment)	$93 Per enrollee-year (0.9% mean total payment)	−$38 Per enrollee-year (−0.4 percentage point)
Aggregate difference in Hispanic-White payment disparities associated with the double-bonus policy (2012-2019)	NA	NA	−$551 Million

Abbreviations: MA, Medicare Advantage; NA, not applicable.

^a^ Regression models and methods for calculating each row are described in the eAppendix in the Supplement. Statistical significance was determined using either the *t* test or the *z* tests and an α level of .05.

## Discussion

In this national study of the MA double-bonus policy, Hispanic enrollees were substantially less likely than White enrollees to live in counties eligible for double bonuses, resulting in lower payments to plans caring for Hispanic populations. Because changes to MA plan payments are partially passed through to enrollees,^6^ bonus disparities likely reduce health care benefits and raise premiums for Hispanic enrollees. Our analysis is limited by the RTI race and ethnicity variable, which undercounts Hispanic enrollees.^5^ Our study also held quality constant to examine structural payment disparities embedded in double bonuses. Given that the average quality performance is worse for Hispanic than White enrollees in MA, the actual payment disparities for plans serving more Hispanic enrollees is likely even larger.^1,2^ As Hispanic enrollment in MA continues to increase, Medicare should eliminate double bonuses and other structural payment disparities in the MA program.

## Notes

Supplement. **eAppendix.** Methods **eFigure.** CONSORT Diagram
eReferences.
