# Supplementary material for: Hispanic-White Differences in Double Bonuses for Quality of Care in Medicare Advantage
Source: JAMA Health Forum. 2022 Mar 4;3(3):e215281. doi: 10.1001/jamahealthforum.2021.5281 (PMC8903100; doi:10.1001/jamahealthforum.2021.5281)
Supplement: Supplement. — eAppendix. Methods eFigure. CONSORT Diagram eReferences. [file jamahealthforum-e215281-s001.pdf]

Supplemental Online Content

Markovitz AA, Montás MC, Warrier A, Ayanian JZ, Ryan AM. Hispanic-White differences in double bonuses for quality of care in Medicare Advantage. *JAMA Health Forum.* 2022;3(3):e215281. doi:10.1001/jamahealthforum.2021.5281

eAppendix. Methods
eFigure. CONSORT Diagram
eReferences.

This supplementary material has been provided by the authors to give readers additional information about their work.

© 2022 Markovitz AA et al. *JAMA Health Forum*.

eAppendix. Methods

Double Bonus Allocation Across Hispanic and White Populations. We used two national data sources from 2012 through 2019 to evaluate how the Centers for Medicare and Medicaid Services (CMS) allocates double bonus eligibility across Hispanic and White populations: the Medicare Beneficiary Summary File and MA ratebook files. The Medicare Beneficiary Summary File was used to define beneficiary enrollment in Medicare Advantage (based on January enrollment each year), age, sex, race/ethnicity (non-Hispanic White or Hispanic), original reason for Medicare entitlement, and dual eligibility for Medicare and Medicaid (for at least one month in the year). MA ratebook files were used to define whether the enrollees resided in a double-bonus county (based on whether the county was eligible in that year). We excluded enrollees with missing covariate data, who resided outside the U.S. or resided within Puerto Rico. We also excluded enrollees who were defined as Hispanic or White (see eFigure for CONSORT diagram)

The Medicare Beneficiary Summary File contains 2 race variables: 1) Medicare enrollment database (EDB) variable, which is based on Social Security Administration (SSA) records, which is known to severely undercount Hispanic beneficiaries[1]; 2) the RTI variable, which imputes missing EDB race data via lists of Hispanic and Asian/Pacific Islander names (from the US Census) and geography (i.e., residence in Puerto Rico or Hawaii). The EDB code undercounts Hispanic beneficiaries for two primary reasons: a) the SSA collects race data from beneficiaries in a voluntary fashion through self-report; b) from 1936 to 1980, the SSA variable included only 3 race codes (White, Black, Other) which was subsequently expanded to 6 codes (non-Hispanic White, non-Hispanic Black, Hispanic, Asian/Asian-American/Pacific Islander American Indian/Alaska Native; Unknown); c) CMS only obtains race information on the retiree and not the retiree's spouse, and subsequently assigns race of the beneficiary to the spouse.[2] Although CMS used multiple strategies to improve race categorization (annual postcard surveys of beneficiaries with Hispanic surnames or countries of birth, use of state Medicaid race/ethnicity data from 32 states), the EDB continues to undercount Hispanic beneficiaries. As a result, the EDB variable is primarily only used for identifying Black and White populations. Although the RTI variable continues to undercount Hispanic beneficiaries somewhat – it is 91% sensitive and 99% specific for predicting Hispanic enrollees, as compared to the gold standard of self-reported race/ethnicity from the OASIS survey[3] – CMS uses the RTI variable in reports on health disparities.[4]

For our analysis of double-bonus exposure among Hispanic versus White MA beneficiaries from 2012 through 2019, we estimated a logistic regression model at the beneficiary-year level. For beneficiary i, residing in county j, and year t, we estimated:

$$(1)\ Double_{ijt} = \beta_1 Hispanic_{it} + \beta_2 X_{it} + \omega_t + v_{ijt}$$

where Double is 1 if the beneficiary's county was eligible for double bonuses in a given year, 0 otherwise; Hispanic is 1 if the enrollee is Hispanic, 0 if non-Hispanic White; X is a vector of beneficiary covariates (age, sex, original reason for Medicare entitlement, Medicaid dual-eligibility); ω represents year fixed effects accounting for annual changes in enrollment; and v is the idiosyncratic error term. We then estimated the difference in the probability of a Hispanic versus White enrollee residing in a double-bonus county via the parameter β_1 on Hispanic. Standard errors were clustered at the county level.

© 2022 Markovitz AA et al. *JAMA Health Forum*.

Impact of Double Bonuses on Payment for MA Plans Caring for Hispanic and White Enrollees. We used three national data sources from 2012 through 2019 to evaluate the impact of double bonus eligibility on payments to MA plans to care for Hispanic and White MA enrollees: the Medicare Beneficiary Summary File, MA Ratebook data, MA Enrollment data, and U.S. Census data. Specifically, we constructed an analytic dataset at the plan-county-year level by linking publicly-available CMS data on plan enrollment, star ratings, and county- or region-specific payment rates. Our analysis focused on Health Maintenance Organization (HMO), local Preferred Provider Organization (PPO), private FFS plans, and regional PPOs, representing 96% of plans from 2012 through 2019 (n=292,898 plan-years, representing 121,766,281 plan-enrollee-years). We excluded MA plans All Cost and Medical Savings Account plans, as payments for these plans are not directly calculated from quality-adjusted county- or region-specific benchmarks. We also excluded low- enrollment plans, as CMS does not report enrollment numbers for these plans and thus we could not include these plans in enrollment-weighted estimates of changes in average and total payments to MA plans. Finally, we excluded plans in Puerto Rico, as these plans are subject to additional payment rules.

We then followed CMS specifications in calculating quality-adjusted payments as a function of star quality ratings and either county-specific benchmarks (for HMOs, local PPOs, and private FFS) or region-specific benchmarks (for regional PPOs). We also followed CMS specifications for determining payments for new plans without star quality ratings.

Using this data set, we first estimated differences in payments to MA plans in double-bonus versus non-double-bonus counties. To do so, we estimated a linear regression model at the plan-county-year level. For plan i, in county j, and in year t, we estimated

$$(2)\ Payment_{ijt} = \beta_1 Double_{jt} + \beta_2 Star_{it} + \beta_3 Benchmark_{jt} + \omega_t + v_{ijt}$$

where Double is 1 if the plan was in a county that was eligible for double bonuses in that year, 0 otherwise; Star is the plan's star rating in that year, Benchmark is the county- or region-specific payment benchmark in that year; ω represents year fixed effects accounting for annual changes in payment; and v is the idiosyncratic error term. We then estimated the enrollment-weighted difference in average per-beneficiary per-year payments for plans in double-bonus versus non-double-bonus counties as the parameter β_1 on Double (or $317.86 per beneficiary-year).

The incremental impact of the double-bonus policy on average payment was then calculated by multiplying the difference in average payment for plans in double-bonus and non-double bonus counties by the probability of residing in double-bonus counties (Hispanic enrollees: $319.83 per enrollee-year x 17.4% = $55.65, White enrollees: $319.83 per enrollee-year x 29.2%=$93.39, a disparity of -$37.74 per-enrollee per-year). This disparity is driven by the fact that Hispanic enrollees are less likely to reside in double bonus counties, as our models control for plans' star rating and county- or region-specific benchmarks, which are the other two determinants of plan payment rates in MA.

The aggregate impact on Hispanic-White payment disparities was then calculated by multiplying the Hispanic-White difference in the incremental effect of the double-bonus policy on payment rates by the number of MA Hispanic enrollees 2012-2019 (-$37.74 per Hispanic enrollee-year x 14,589,670 enrollees-years=$551m).

© 2022 Markovitz AA et al. *JAMA Health Forum*.

eFigure. CONSORT Diagram

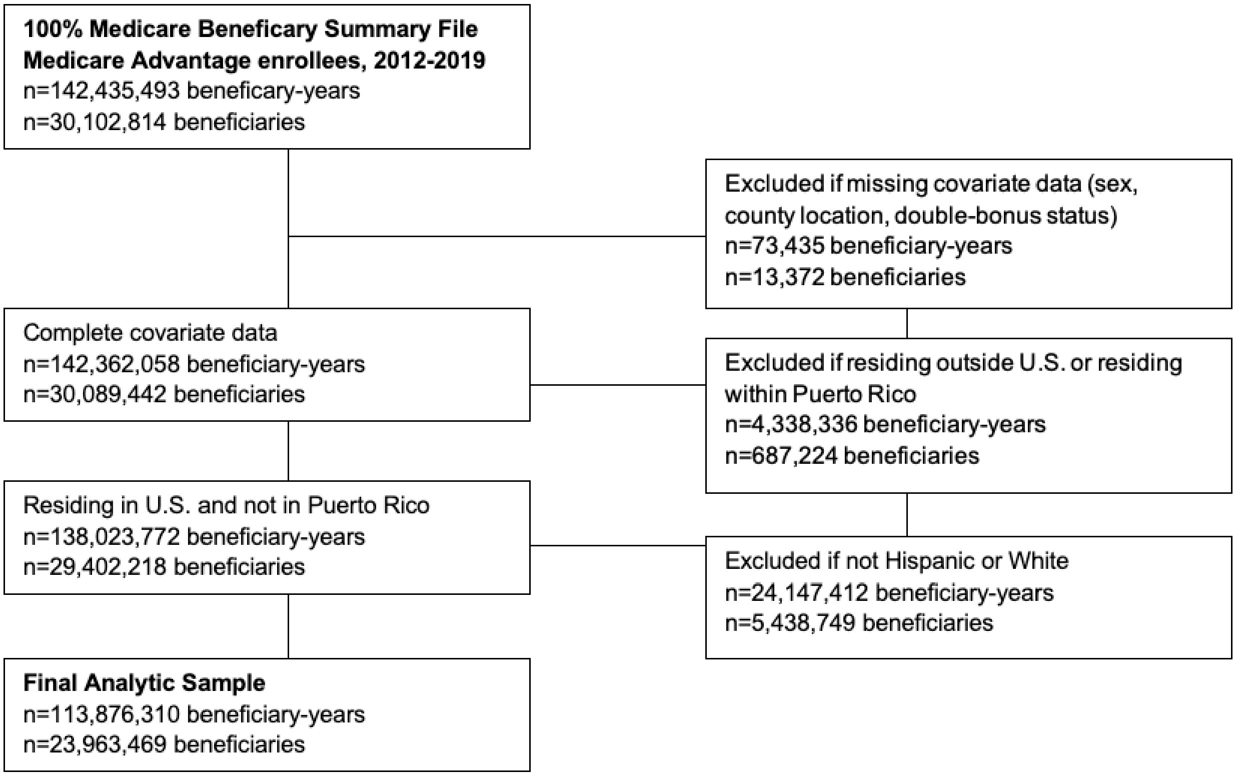

© 2022 Markovitz AA et al. *JAMA Health Forum*.

eReferences

1. Filice CE, Joynt KE. Examining Race and Ethnicity Information in Medicare Administrative Data. *Medical Care*. 2017;55(12). doi:10.1097/MLR.0000000000000608
2. National Research Council. *Eliminating Health Disparities: Measurement and Data Needs*.; 2004.
3. Jarrín OF, Nyandege AN, Grafova IB, Dong X, Lin H. Validity of race and ethnicity codes in Medicare administrative data compared with gold-standard self-reported race collected during routine home health care visits. *Medical Care*. 2020;58(1). doi:10.1097/MLR.0000000000001216
4. Centers for Medicare & Medicaid Services Office of Minority Health. *The Mapping Medicare Disparities Tool Technical Documentation*.; 2021. Accessed December 21, 2021. https://www.cms.gov/About-CMS/Agency-Information/OMH/Downloads/Mapping-Technical-Documentation.pdf

© 2022 Markovitz AA et al. *JAMA Health Forum*.
